# Using vaginal discharge score (VDS) grading system to evaluate the effect of clinical endometritis on reproductive performance of dairy cows in China

**DOI:** 10.1590/1984-3143-AR2020-0228

**Published:** 2021-05-14

**Authors:** Hongsheng Wang, Zuoting Yan, Xiaohu Wu, Yong Zhang, Yubing Wei, Xingxu Zhao

**Affiliations:** 1 College of Veterinary Medicine, Gansu Agricultural University, Lanzhou, China; 2 Gansu Key Laboratory of Animal Generational Physiology and Reproductive Regulation, Lanzhou, China; 3 Lanzhou Institute of Husbandry and Pharmaceutical Sciences of Chinese Academy of Agricultural Sciences, Lanzhou, China; 4 The Animal Husbandry and Veterinary Station of Ganzhou County, Zhangye, China

**Keywords:** clinical endometritis, vaginal discharge score, diagnosis, reproductive performance

## Abstract

Clinical endometritis (CE) is a major cause in affecting the reproductive performance of dairy cows. The objectives of this study were to ascertain the prevalence of CE and to evaluate the effect of CE on reproductive performance in dairy cows using vaginal discharge score (VDS) grading system. 803 dairy cows were examined by vaginoscope with 4-point VDS at 26 ± 3 days in milk (DIM) and classified into six groups: non-endometritis with VDS 0 (control; CON), endometritis with VDS 1 (MEM), non-treated endometritis with VDS 2 (NTME), treated endometritis with VDS 2 (TME), non-treated endometritis with VDS 3 (NTPE), and treated endometritis with VDS 3 (TPE). Cows in TME and TPE groups were treated with 200 mL of 50% dextrose solution by intrauterine infusion. The prevalence of CE was 33% at 26 ± 3 DIM. Binary logistic regression analysis revealed cows in MEM, NTME and NTPE groups had a less likelihood of first artificial insemination (AI) pregnancy than those in CON group (*P* < 0.05). Kaplan-Meier survival curves for days open were statistically different (*P* = 0.004). In Cox regression model, cows in NTME and NTPE groups had a reduced pregnancy rate than those in CON group (*P* < 0.05). The hazard of pregnancy in NTME group was lower than that in TME group (*P* = 0.044). Similarly, it was lower for the hazard of pregnancy in NTPE group than in TPE group (*P* = 0.048). Cows in MEM, NTME, and NTPE groups required more services per pregnancy than those in CON group (*P* < 0.05). In conclusion, CE examined by the VDS grading system impaired reproductive performance, and mild endometritis with VDS 1 should be treated in the early postpartum period to ameliorate fertility in dairy herds.

## Introduction

Reproductive performance is a key factor in determining the profitability of dairy production management (Fourichon et al., 2000; LeBlanc et al., 2002a). Clinical endometritis (CE) is a common cause of reduced reproductive efficiency in dairy cows, which is characterized by the presence of mucopurulent or purulent uterine exudate in the vagina for 21 days or more post partum, and without systemic signs (Sheldon et al., 2006; Dubuc et al., 2010a). The morbidity of CE in dairy cows ranges from 6.7% to 47.0% (Bicalho et al., 2010; Dubuc et al., 2011). Several studies have suggested that multiple risk factors for CE include perineum hygiene at the time of calving, peripartum metabolic status, retained placenta, dystocia, twins, and parity (Földi et al., 2006; LeBlanc, 2008; Sheldon et al., 2009; Dubuc et al., 2010b; Schuenemann et al., 2011). Diagnosis and treatment of CE, and its potential impact on reproductive performance, have attracted considerable attention from dairy farmers and veterinary practitioners.

Techniques used to diagnose endometritis include transrectal palpation, a gloved hand, ultrasonography, vaginoscopy, the Metricheck device, endometrial biopsy, and endometrial cytology (Bonnett et al., 1993; Kasimanickam et al., 2004; McDougall et al., 2007; Pleticha et al., 2009; Dubuc et al., 2010a; Leutert et al., 2012), which allow a given prognosis for impaired subsequent fertility. Endometrial biopsy is the perfect method to diagnose endometritis and has been associated with a detrimental effect on fertility (Bonnett et al., 1991), but this technique is expensive and time-consuming, and not clinically accessible in most cases (Sheldon et al., 2006). Endometrial cytology is more practical and has been suggested as the definitive diagnosis of subclinical endometritis (Kasimanickam et al., 2004). A previous study suggested cervical diameter greater than 7.5 cm diagnosed by transrectal palpation was used as an additional predictive value for decreased fertility (LeBlanc et al., 2002b). However, the sensitivity and specificity of this method are low for endometritis diagnosis (LeBlanc, 2003; Dubuc et al., 2011) and its findings have little association with fertility (Sheldon et al., 2006; McDougall, 2010).

In clinical practice, an accurate diagnosis of CE is based on the presence of uterine pus in the vaginal discharge (Sheldon and Noakes, 1998; LeBlanc et al., 2002b). A common method used to examine vaginal discharge is vaginoscopy based on vaginal discharge score (VDS) (LeBlanc et al., 2002b; Williams et al., 2005; Sheldon et al., 2006). The VDS grading system was established by Williams et al. (2005) and vaginal discharge was scored using a 0 to 3 scale: VDS 0 = no or clear mucus (non-endometritis); VDS 1 = discharge containing flecks of white or off-white pus (mild endometritis); VDS 2 = discharge containing less than 50% white or off-white mucopurulent pus (mucopurulent endometritis); and VDS 3 = discharge containing more than 50% white or yellow purulent pus (purulent endometritis). The character score of vaginal pus can well reflect the presence of certain bacteria in the uterus, whereby cows with higher VDS were more likely to have *A. pyogenes*, *F. necrophorum* and *Proteus* species (Huszenicza et al., 1999; Williams et al., 2005; Westermann et al., 2010). Mucopurulent or purulent endometritis detected by vaginoscopy has been associated with reduced reproductive performance (LeBlanc et al., 2002b). Mild endometritis is not associated with the presence of higher numbers of pathogenic bacteria in the uterus, but the effects on the reproductive performance of dairy cows are still controversial, particularly whether cows with VDS 1 require treatment or not (LeBlanc et al., 2002b; Williams et al., 2005; Barlund et al., 2008; Leutert et al., 2012; Okawa et al., 2017). Although the VDS grading system has been applied to diagnose CE and to estimate reproductive prognosis in dairy cows in western industrialized countries, it is still disputable whether the findings of this method can predict the reproductive performance in dairy herds in other countries or regions because of differences in herd health management, environment and breeding levels. The information regarding its effectiveness in the assessment of reproductive performance in dairy herds in China is limited. Hence, the main objectives of this study were 1) to ascertain the prevalence of CE, and 2) to evaluate the effect of CE on subsequent reproductive performance of dairy herds using the VDS grading system in China. Our hypothesis was that the VDS grading system based on the characteristics of uterine pus in the vagina could better anticipate fertility.

## Materials and methods

### Animals and feeding management

This study was conducted between October 2018 and October 2019 in three commercial herds with similar management practices in the Zhangye region of northwest China (100°27'21” E; 38°56'11” N). In total, 803 lactating Holstein cows (256 cows in herd A, 281 cows in herd B, 266 cows in herd C) were examined for CE at 26 ± 3 DIM. The parity ranged from one to seven. All cows were housed in free-stall barns and bedded with sand. Additionally, all cows were non-seasonal, milked three times daily at approximately eight hours intervals. The average milk production varied between 8,020 and 10,100 kg/cow/year, with 4.2 percent fat and 3.4 percent protein. In three herds, cows were fed twice daily with a diet of total mixed ration consisting of corn silage, alfalfa, concentrates and had free access to fresh water. Particularly, the voluntary waiting period (VWP) in each herd was set at 40 days postpartum.

### Experimental design

Biweekly, a list of dairy cows at 26 ± 3 DIM was obtained from on-farm computer records and each herd was visited on a fixed day. Briefly, all cows were restrained using headlocks within the pen immediately after milking for assessment of body condition score (BCS) and diagnosis of CE. Once the cow was in the headlock, BCS was examined by an investigator for changes along a 1 to 5 scale, using .25-unit increments (Edmonson et al., 1989). For this visual technique, a score of 1 indicated an emaciated condition, and a score of 5 indicated an obese condition. And then, the tail of cow was held to one side by a farmer, the perineum area was thoroughly wiped with dry paper to remove fecal material and disinfected with 75% ethanol and vaginoscope (Hauptner and Herberholz GmbH & Co. KG, Solingen, Germany; length: 30 cm, diameter: 2.2 cm) was moistened with 0.9% sodium chloride solution, and inserted into the vagina up to the level of the external os of the cervix. The cervix and vagina were visually inspected for the presence and quality of discharge with the help of a flashlight (Leutert et al., 2012). The examination lasted for 10 to 30 s/cow. The character of vaginal mucus was scored with VDS 0 to 3 and recorded. Cows diagnosed with VDS 0 were considered healthy and cows diagnosed with VDS 1 to 3 were considered to have CE. Both BCS and the vaginoscopy were performed by the same investigator.

**Figure 1:**
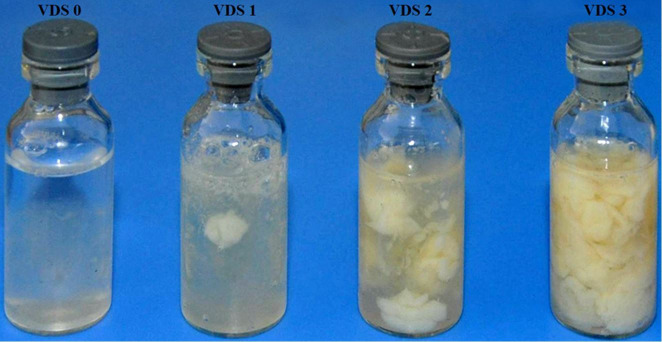
Typical samples of vaginal mucus character score. VDS 0: no or clear mucus; VDS 1: discharge containing flecks of white or off-white pus; VDS 2: discharge containing less than 50% white or off-white mucopurulent pus; and VDS 3: discharge containing more than 50% white or yellow purulent pus.

To determine the impact of CE on reproductive performance, according to the vaginal mucus character scores (Figure 1), cows were allocated randomly into six groups: non-endometritis with VDS 0 (control; CON group); non-treated, mild endometritis with VDS 1 (MEM group); treated, mucopurulent endometritis with VDS 2 (TME group); non-treated, mucopurulent endometritis with VDS 2 (NTME group); treated, purulent endometritis with VDS 3 (TPE group); and non-treated, purulent endometritis with VDS 3 (NTPE group). Clinically healthy cows with VDS 0 received no placebos. Cows with VDS 1 received no further treatment. Cows with VDS 2 or 3 were subdivided into subgroups: one was treated with intrauterine infusion of 200 mL of 50% dextrose solution (Sheng’Ao Animal Pharmaceutical Co., Ltd. Shanxi, China), whereas the other subgroup was treated without intervention. The details of the treatment protocol of dextrose and its effects were reported elsewhere (Brick et al., 2012; Maquivar et al., 2015; Ahmadi et al., 2019). After 14 days therapy, vaginal discharge was re-evaluated at 40 ± 3 DIM using the same VDS grading system previously described. Cows that had no or clear mucus discharge (VDS 0) were considered to recover from CE. After treatment and re-evaluation, all cows followed the normal herd reproductive management practices in the breeding period.

**Figure 2:**
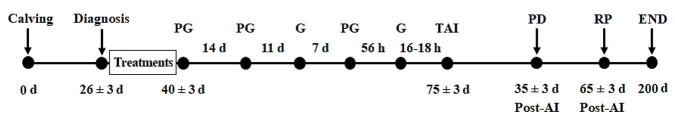
Scheme of the experimental design. PG: prostaglandin; G: GnRH; TAI: Timed artificial insemination; PD: Pregnancy diagnosis; RP: Reconfirmation of pregnancy; END: Pregnancy until 200 DIM.

Reproductive management started at 40 days after postpartum, all cows were received intramuscular injection of a dose of PGF2α (25 mg, Lutalyse; Zoetis Belgium SA). During the following 14 days, estrus was detected once daily and those showing signs of standing estrous behavior were received artificial insemination (AI). Cows that were not in estrus received a second dose of PGF2a 14 days later and were inseminated 12 hours after estrus detection. Eleven days after the second injection of PGF2α, cows that still did not display estrous behavior were enrolled in a timed-AI (TAI) protocol. The initial intramuscular application of GnRH (100 μg, Gonadorelin; Sansheng Biological Technology Co., Ltd. Ningbo, China) was followed 7 days later by an injection of PGF2α and 56 hours later, cows were administered the second dose of GnRH and were timed artificially inseminated 16 to 18 hours later (Figure 2). Additionally, non-pregnant dairy cows at the time of pregnancy diagnosis were re-enrolled in a TAI protocol as described previously. Pregnancy diagnosis was made 35 ± 3 d post-AI via transrectal ultrasonography and reconfirmation of pregnancy was made 65 ± 3 d post-AI.

The following outcomes were measured to assess reproductive performance: days to first AI (interval from calving to first artificial insemination), first AI pregnancy rate (number of cows pregnant after first AI divided by the number of cows inseminated × 100), service per pregnancy (the average number of artificial inseminations until pregnancy), days open (interval from calving to pregnancy), and pregnancy rate (number of cows documented to be pregnant by 200 DIM divided by the number of cows enrolled × 100). All diagnoses and treatments were documented in the case of reported forms. At the end of the study, reproductive data of individual dairy cows were collected until at least 200 days after parturition.

### Statistical analysis

Data were analyzed using SPSS (version 22.0, IBM SPSS Statistics; New York, USA). An exploratory analysis was initially performed to ascertain the incidence of CE of dairy cows diagnosed with VDS of 1 to 3 at 26 ± 3 DIM. A binary logistic regression was obtained to evaluate parity and BCS as risk factors for CE. The results for days to first AI and service per pregnancy of the studied cows were analyzed using Kruskal-Wallis test. A binary logistic regression was utilized to estimate the risk of pregnancy after first AI. In the models, days to first AI pregnancy was dependent variable and the following were considered as independent variables: treatments, parity and BCS. Kaplan - Meier test was obtained to estimate days open in different treatments and the equality of different survival curves was compared by the log-rank test. A Cox regression was fitted to estimate the hazard of pregnancy within 200 DIM. In the models, the time variable was the interval in days from calving to pregnancy and the explanatory variable included treatments, parity and BCS. BCS at 26 ± 3 DIM was classified as ≤ 2.75 and ≥ 3.0; parity was classified as primiparous and multiparous. Observations were censored, if cows that were not pregnant or never inseminated at 200 DIM. We reported adjusted odds ratios (OR), hazard ratios (HR), confidence intervals (CI) and *P* values. In this study, CI was set at 95% for logistic regression and survival analyses, and *P* < 0.05 was considered significant for all statistical analyses.

This study was approved by the Ethics Committee for Animal Experimentation of the Gansu Agricultural University (protocol number 29/2018).

## Results

Initially, 803 enrolled dairy cows were screened for CE at 26 ± 3 DIM, and data from 42 cows were excluded from the analysis because cows were culled or died from other causes of illness (n = 31), or any other incomplete information that may influence the results (n = 11) during the study period. Finally, 761 enrolled dairy cows were available for further statistical analysis of reproductive performance, with 506 (66.5%) being classified as CON group, 99 (13%) as MEM group, 39 (5.1%) as NTME group, 45 (5.9%) as TME group, 34 (4.5%) as NTPE group, and 38 (5%) as TPE group; 268 for primiparous cows and 493 for multiparous cows; 264 for cows with BCS ≤ 2.75 and 497 for cows with BCS ≥ 3.0.

### Prevalence of endometritis

The prevalence of CE diagnosed by the VDS grading system was shown in Table 1. The overall prevalence of CE was 33% at 26 ± 3 DIM in three commercial dairy herds, of which VDS 1, VDS 2 and VDS 3 was 12.9%, 10.8% and 9.3%, respectively. The incidence of CE was equivalent in individual herds, which was 34%, 36.3%, and 28.9%, respectively. The parity proved to be a risk factor (*P* < 0.001) for endometritis, with OR of 1.765, suggesting that the chance of a multiparous cow was 1.765 times higher to present endometritis than a primiparous cow. There was no significant difference in endometritis between cows with BCS ≤ 2.75 and cows with BCS ≥ 3.0 (OR = 1.286, *P* = 0.114).

**Table 1:** Prevalence (%) of different degrees of VDS of dairy cows at 26 ± 3 DIM in three commercial dairy herds considering parity and BCS.

**Outcome**	**VDS 0**	**VDS 1**	**VDS 2**	**VDS 3**
Herds				
A, n = 256	66.0	13.7	10.9	9.4
B, n = 281	63.7	14.2	11.7	10.3
C, n = 266	71.1	10.9	9.8	8.3
Parity				
Primiparous, n = 288	74.7	10.8	7.3	7.3
Multiparous, n = 515	62.5	14.2	12.8	10.5
BCS				
≤ 2.75, n = 284	70.4	12.7	8.1	8.8
≥ 3.0, n = 519	64.9	13.1	12.3	9.6

VDS 0: no or clear mucus; VDS 1: discharge containing flecks of white or off-white pus; VDS 2: discharge containing less than 50% white or off-white mucopurulent pus; VDS 3: discharge containing more than 50% white or yellow purulent pus; BCS: Body condition score.

### Effect of CE categorized using VDS grading system on reproductive performance

Binary logistic regression analysis for the likelihood of the first AI pregnancy rate was shown in Table 2. The first AI pregnancy rate in CON group was 38.3%, 26.2% for MEM, 17.1% for NTME, 34.8% for TME, 16.7% for NTPE, and 33.3% for TPE. Cows in MEM, NTME, NTPE groups had a less likelihood of first AI pregnancy rate compared with cows in CON group (OR = 0.601, *P* = 0.037; OR = 0.409, *P* = 0.029; OR = 0.314, *P* = 0.012, respectively), while there were no significant differences between the other groups. Additionally, the first AI pregnancy rate was 44.4% and 31.6% in primiparous cows and multiparous cows, respectively. Multiparous cows had an OR for the first AI pregnancy of 56.3% (*P* < 0.001), or a 44.7% reduction in the first AI pregnancy rate than primiparous cows. The first AI pregnancy rate was higher for cows with BCS ≥ 3.0 than cows with BCS ≤ 2.75 (OR = 1.481, *P* = 0.020). The days to first AI were 73.4 ± 13.5 days, 76.1 ± 18 days, 78.4 ± 19.1 days, 74.5 ±11.9 days, 78.5 ± 16.8 days, and 73.6 ± 14.8 days in CON, MEM, NTME, TME, NTPE and TPE groups, respectively. There was no significant difference for days to first AI between the groups (*P* = 0.267).

**Table 2:** Results of binary logistic regression analysis for the likelihood of first AI pregnancy in dairy cows examined for CE at 26 ± 3 DIM, including treatments, parity, and BCS as covariates.

Explanatory variable			Pregnancy after first AI		
OR		95% CI		*P*-value	
Treatments					
CON	Reference		Reference		Reference
MEM	0.601		0.373-0.969		0.037
NTME	0.409		0.183-0.914		0.029
TME	0.821		0.432-1.563		0.549
NTPE	0.314		0.127-0.778		0.012
TPE	0.829		0.410-1.677		0.601
Parity	0.563		0.409-0.776		< 0.001
BCS	1.481		1.063-2.062		0.020
Constant		0.741		0.057	

AI: artificial insemination; OR: Odds Ratios; CI: Confidence Intervals; *P*: Probability; CON: non-endometritis with VDS 0; MEM: mild endometritis with VDS 1; NTME: non-treated, mucopurulent endometritis with VDS 2; TME: treated, mucopurulent endometritis with VDS 2; NTPE: non-treated, purulent endometritis with VDS 3; TPE: treated, purulent endometritis with VDS 3; BCS: Body condition score.

**Figure 3:**
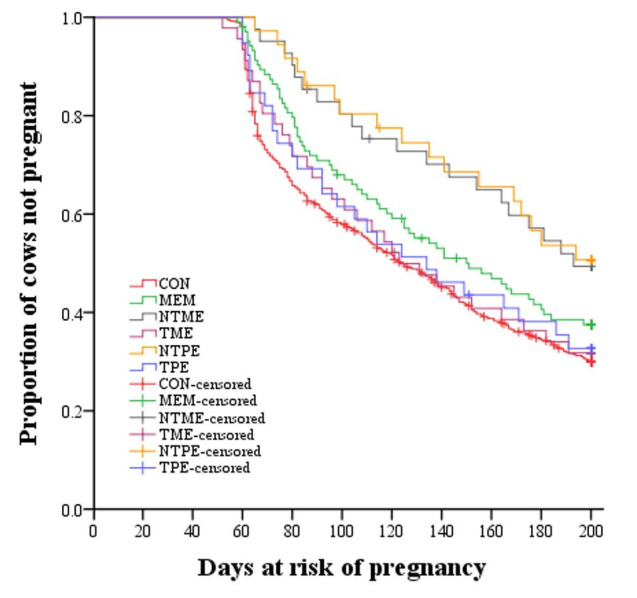
Kaplan-Meier survival curves for time to pregnancy (days open) for dairy cows with different treatments.

Kaplan-Meier survival curves for days open of different treatment groups were shown in Figure 3. The survival curves in different treatments were statistically different (*P* = 0.004). The survival time of pregnancy (days open) of 25% of CON group was 67 days (n = 506, 5.8% censored), 83 days (n = 99, 4.8% censored) for MEM, 122 days (n = 39, 5.1% censored) for NTME, 79 days (n = 45, 2.2% censored) for TME, 124 days (n = 34, 5.9% censored) for NTPE, and 74 days (n = 38, 2.6% censored) for TPE, indicating a 55-day delay to achieve the same percentage of pregnant cows in NTME group compared to CON group, 57-day delay in NTPE compared to CON group, 43-day delay in NTME compared to TME group, 50-day delay in NTPE compared to TPE group.

The results of Cox regression analysis for the hazard of pregnancy within 200 DIM were shown in Table 3. The pregnancy rate of cows in CON group was 72.1%, 63.6% for MEM, 51.3% for NTME, 68.9% for TME, 50.0% for NTPE, and 68.4% for TPE. Cows in NTME group had a HR for the pregnancy of 0.558 (*P* = 0.011), or a 44.2% reduction in pregnancy rate relative to those in CON group. Cows in NTPE group had a reduced pregnancy rate than those in CON group (HR = 0.516, *P* = 0.008). The hazard of pregnancy in NTME group was lower than that in TME group (HR = 0.558, *P* = 0.044). Similarly, it was lower for the hazard of pregnancy in NTPE group than in TPE group (HR = 0.533, *P* = 0.048). The hazard of pregnancy in the CON group was not significantly different from the other groups. Additionally, The pregnancy rate was 84.3% and 60% in primiparous cows and multiparous cows, respectively. The pregnancy regression coefficient showed that the hazard of pregnancy for multiparous cows was only 56.4% of that of primiparous cows (HR = 0.564, *P* < 0.001). The factor BCS showed no significant difference of pregnancy between levels (HR = 1.106, *P* = 0.283). Services per pregnancy were 1.8 ± 1.1 for CON group, 2.2 ± 1.4 for MEM, 2.4 ± 1.4 for NTME, 2 ± 1.3 for TME and 2.5 ± 1.5 for NTPE, and 1.9 ± 1.1 for TPE. Cows in MEM, NTME and NTPE groups required a significantly higher service per pregnancy to become pregnant as compared to CON group (*P* = 0.014; *P* = 0.04 and *P* = 0.035, respectively).

**Table 3:** Results of Cox regression analysis for the hazard of pregnancy at 200 DIM in dairy cows examined for CE at 26 ± 3 DIM, including treatments, parity, and BCS as covariates.

Explanatory variable	Pregnancy until 200 DIM		
	HR	95% CI	*P*-value
Treatments			
CON	Reference	Reference	Reference
MEM	0.808	0.618-1.057	0.120
NTME	0.558	0.355-0.877	0.011
TME	0.953	0.659-1.378	0.799
NTPE	0.516	0.317-0.840	0.008
TPE	0.927	0.622-1.380	0.708
Parity	0.564	0.471-0.676	< 0.001
BCS	1.106	0.920-1.329	0.283

DIM: Days in milk; HR: Hazard Ratios; CI: Confidence Intervals; *P*: Probability; CON: non-endometritis with VDS 0; MEM: mild endometritis with VDS 1; NTME: non-treated, mucopurulent endometritis with VDS 2; TME: treated, mucopurulent endometritis with VDS 2; NTPE: non-treated, purulent endometritis with VDS 3; TPE: treated, purulent endometritis with VDS 3; BCS: Body condition score.

## Discussion

This study is one of the few trials to ascertain the prevalence of CE and to investigate the association between CE and subsequent reproductive performance in postpartum dairy cows using the VDS grading system in China. The overall prevalence of CE in the present study (33%) was almost similar to that in the previous study (36.8%) by Pleticha et al. (2009) using vaginoscopy conducted under comparable housing conditions but slightly lower than other previous studies (42.6%) (Leutert et al., 2012). In our study, VDS 1 was defined as mild endometritis and included in the overall prevalence of endometritis, which was consistent with some previous studies (Pleticha et al., 2009; Okawa et al., 2017), but other studies defined VDS 1 as healthy (LeBlanc et al., 2002b; Barlund et al., 2008).

The VDS 2 and 3 were explicitly defined as having a certain percentage of uterine pus in vaginal discharge, whereas VDS 1 was not specified a specific percentage (Williams et al., 2005). Previous studies suggested cows with a VDS 1, 2, and 3 were classified as having CE and required treatment (Pleticha et al., 2009; Okawa et al., 2017). However, other studies showed that cows with VDS 1 were not being associated with reduced pregnancy rate and thus as healthy (LeBlanc et al., 2002b; Barlund et al., 2008; Westermann et al., 2010). A publication by Gautam et al. (2010) also suggested that the presence of mild endometritis with VDS 1 did not have a detrimental effect on reproductive performance, and that the most cases of mild endometritis might recover spontaneously by the physiologic self-healing capacity of the uterus. It is unknown whether cows with VDS 1 should require treatment or not. Based on the previous literature, we hypothesized that cows with VDS 1 had the ability to recover spontaneously and need no further intervention. In our observations, however, cows with VDS 1 had a lower pregnancy rate after first AI and required more services per pregnancy as compared to the healthy cows. The effect of CE on reproductive performance was also evaluated by Okawa et al. (2017). In their study, cows with VDS 1 that did not receive treatment required a significantly higher number of AI to become pregnant and showed lower reproductive efficiency. The absence of uterine pus in the vagina does not always reflect the absence of infection in the uterus and normal reproductive function (Sheldon et al., 2006). Based on the current results, we speculate that some cases diagnosed with VDS 1 may fail to fully recover through the self-healing ability of the uterus, and may have developed into persistent mild endometritis or severe endometritis, which caused decreased fertility. Furthermore, since there is no golden standard for determining endometritis in clinical practice in this field, some cows classified as having mild endometritis by the VDS grading system may have had subclinical endometritis (Okawa et al., 2017). Previous studies provided clear evidence that subclinical endometritis is characterized by the absence of pus in the vagina at the early stage, and is related to the decrease in pregnancy rate after first AI and overall (Kasimanickam et al., 2004; Gilbert et al., 2005). In the present study, however, cows with VDS 1 were not further examined for subclinical endometritis. Thus, we should consider the existence of subclinical endometritis in the early postpartum period when evaluating the effects of endometritis detected by the VDS grading system on reproductive performance in clinical practice. Overall, we hold the opinion that mild endometritis with VDS 1 has a negative impact on the reproductive performance of dairy cows in the early postpartum period and therefore requires treatment to ameliorate uterine health and fertility.

The present study confirmed findings that endometritis with VDS 2 or 3 has a significant adverse impact on subsequent fertility and the reproductive efficiency in dairy cows decreased with an increasing VDS. The substantial impairments of reproductive performance were reflected in the reduction in the first AI pregnancy rate, the delay in open days, the increase in services per pregnancy and the reduction in pregnancy rates by the end of the study, which were in alignment with previous observations (LeBlanc et al., 2002b; McDougall et al., 2007; Gautam et al., 2009). In our study, 26 ± 3 DIM was set as the standard days for evaluating the impact of endometritis on reproductive performance, whereas in other studies, different postpartum intervals were selected as the evaluation days. LeBlanc et al. (2002b) showed that cows with VDS 2 and 3 took 27% longer to become pregnant than cows without endometritis between 20 and 33 DIM. In other study, cows with clinically relevant endometritis were always associated with reduced pregnancy rate within 60 days postpartum period (Gautam et al., 2009). Furthermore, McDougall et al. (2007) reported that cows with VDS 2 or more had a lower probability of pregnancy after first AI and took longer to conceive at 35 days before the start of the seasonal breeding programme. It is obvious that the different postpartum intervals of diagnosis at examinations don’t alter the negative impacts of CE on reproductive performance. Therefore, we suggest that affected cows require treatment to improve reproductive efficiency when endometritis with VDS 1 to 3 is detected in dairy cows using the VDS grading system at any time during the postpartum period.

The treatment employed here was the intrauterine infusion of a hypertonic solution of 50% dextrose in cows with VDS 2 and 3. Some researchers believed that utilizing the hypertonic solution of 50% dextrose as a non-antibiotic treatment in dairy cows with endometritis improved uterine health by inhibiting local bacterial growth, increasing uterine tone, reducing the water activity of bacteria and nurturing endometrial cells (Brick et al., 2012; Maquivar et al., 2015; Ahmadi et al., 2019). In the present study, cows diagnosed with VDS 2 and 3 that were treated with dextrose increased pregnancy rate at 200 DIM, and shortened intervals in achieving the same percentage of pregnancy as compared to those that were untreated, which had a very similar reproductive performance to healthy cows. Brick et al. (2012) reported that intrauterine infusion of 50% dextrose increased the first AI pregnancy rate of CE cows compared with receiving ceftiofur hydrochloride subcutaneously behind the ear. A randomized clinical study showed that administration of dextrose in cows with mucopurulent or purulent vaginal discharge alone improved the proportion of clinical cure, resumption of postpartum estrous cycle, and pregnancy per AI compared with untreated cows at 26 ± 3 DIM (Maquivar et al., 2015). A study showed that days open and pregnancy rate before 200 DIM of CE cows treated with dextrose were equivalent to those of healthy cows (Ahmadi et al., 2019). However, a previous study had also reported the potential effects of dextrose infusion on the cure rate and reproductive performance of dairy cows during lactation (Machado et al., 2015). Unlike our findings and other studies, they reported that intrauterine infusion of 50% dextrose solution was a noneffective approach for curing CE and had no effect on first AI conception rate, pregnancy maintenance and calving to conception interval. The difference in these observations regarding the effect of dextrose infusion on the reproductive performance of dairy cows may be caused by the difference of diagnostic criteria for CE based on the VDS grading system. In Brick et al. (2012) study, they observed the vaginal discharge diagnosed using the vaginoscopy with a VDS 0 to 3 scale that did not take into account vaginal contents composed of less than 50% purulent content, which was similar to the gloved hand technique employed in previous studies by Maquivar et al. (2015) and Ahmadi et al. (2019), whereas in the study by Machado et al. (2015), the vaginal discharge was scored by a Metricheck device with a VDS 0 to 5 scale and any presence of pus beyond flecks in vaginal discharge was diagnosed as CE. Despite that, our current study suggests administration of intrauterine dextrose improves the reproductive performance of dairy cows with CE.

The incidence of endometritis and the reproductive performance of dairy cows are significantly associated with parity and BCS. There are differences in the reviews regarding the effects of parity on the incidence of endometritis. Previous studies showed there was no association between the parity and prevalence of endometritis (Gilbert et al., 2005; Hendricks et al., 2006). In contrast, Runciman et al. (2008) found the prevalence of endometritis was greatly associated with cows in primiparous parity. However, in present study, multiparous cows at examination were associated with a higher risk of endometritis; similar results were shown by previous studies (LeBlanc et al., 2002b; Machado et al., 2015). The cause for the higher prevalence of endometritis in multiparous cows is not clear. The possible explanation is that an increased incidence of endometritis may be related to an impairment of neutrophil function with age (Lee and Kim, 2006). In addition, we observed that multiparous parity at examination was associated with reduced pregnancy rate after first AI and reduced pregnancy rate at 200 DIM, which was consistent with the results of Pleticha et al. (2009). In the present study, there was no significant effect of BCS on the prevalence of endometritis, consistent with the results of previous studies (LeBlanc et al., 2002b; Gautam et al., 2009). However, the increased risk of endometritis was associated with a low BCS in seasonally calving dairy herds (Runciman et al., 2008). There was a significant reduction in pregnancy rate after first AI in cows with low BCS, which was in accordance with the results of McDougall et al. (2013). However, BCS at examination was not associated with a reduction in pregnancy rate at 200 DIM. One possible explanation is that this is related to increased energy intake in dairy cows during lactation management, which may be conducive to improving reproductive efficiency. Our current results indicate that the higher incidence of endometritis is associated with multiparous parity and the reduction in reproductive performance is associated with multiparous parity and low BCS.

The difficulty in assessing the impacts of CE on reproductive performance lies in achieving higher accuracy of diagnosis. In the field, any diagnostic methods based on vaginal discharge for endometritis are unlikely to reach 100% sensitivity and specificity when measuring reproductive performance because of other unknown, independent reasons. Metricheck device as a novel vaginal examination method for diagnosis of CE has higher sensitivity, but its specificity is lower than that of vaginoscopy (McDougall et al., 2007). Leutert et al. (2012) reported the sensitivity and specificity of vaginoscopy with VDS were 99.6 and 96.7%, 96.3 and 90.1% in two experiments, respectively, when VDS 0 was considered as healthy and VDS 1 to 3 as CE. However, in a study by Westermann et al. (2010) showed that the false positive proportion of CE examined by vaginoscopy in dairy cows was 17.3% to 28.5% when intrauterine bacteriology and cytology were used as the reference diagnostic tests. Although vaginoscope is defective (Gautam et al., 2010; Leutert et al., 2012), it is still a practical method for anticipating reproductive performance in dairy cows under field conditions. In our study, all cows were examined only once by vaginoscopy; visual assessment of the VDS grading system might cause the errors in defining vaginal discharge. The presence of VDS more than 1 in dairy cows in the present study may be incorrectly classified as VDS 0 or 1. This could be attributed to the reduced field of vision provided by the vaginoscope and the limited brightness of the flashlight (Leutert et al., 2012). Therefore, we suggest endometritis diagnosed with VDS 0 or 1 using the VDS grading system should be reassessed at clinical examinations to improve the sensitivity of the diagnosis process.

## Conclusion

The effect of endometritis with different degrees of VDS on reproductive performance in dairy cows varies from region to region. 33% of dairy cows showed VDS 1 to 3 at 26 ± 3 DIM and had reduction in subsequent reproductive performance in China. The decision whether cows with different degrees of VDS need to be treated should depend on the degree of uterine infections and the status of subsequent reproductive performance. Treatment for VDS 1 in the early postpartum period is beneficial to ameliorate the fertility of dairy cows.
